# Computational perspectives on human fear and anxiety

**DOI:** 10.1016/j.neubiorev.2022.104959

**Published:** 2023-01

**Authors:** Yumeya Yamamori, Oliver J. Robinson

**Affiliations:** a Institute of Cognitive Neuroscience, University College London, UK; b Clinical, Educational and Health Psychology, University College London, UK

**Keywords:** Anxiety, Fear, Computational modelling, Generative models, Reinforcement learning, Approach-avoidance conflict, Uncertainty, Decision-making

## Abstract

Fear and anxiety are adaptive emotions that serve important defensive functions, yet in excess, they can be debilitating and lead to poor mental health. Computational modelling of behaviour provides a mechanistic framework for understanding the cognitive and neurobiological bases of fear and anxiety, and has seen increasing interest in the field. In this brief review, we discuss recent developments in the computational modelling of human fear and anxiety. Firstly, we describe various reinforcement learning strategies that humans employ when learning to predict or avoid threat, and how these relate to symptoms of fear and anxiety. Secondly, we discuss initial efforts to explore, through a computational lens, approach-avoidance conflict paradigms that are popular in animal research to measure fear- and anxiety-relevant behaviours. Finally, we discuss negative biases in decision-making in the face of uncertainty in anxiety.

## Highlights

• There is increasing interest in computational approaches to human fear and anxiety.
• We focus on ‘generative models’ of fear-/anxiety-related behaviours.
• Key themes of aversive learning, approach-avoidance conflict, and uncertainty.
• We discuss candidate computational mechanisms of pathological fear/anxiety.

## 1 Introduction

Fear and anxiety are adaptive states which elicit defensive behaviours that help organisms avoid harm and ultimately survive (LeDoux and Pine, 2016). They are commonly distinguished in the literature on the basis of the immediacy, proximity and/or certainty of the threat, with fear and fear-related responses evoked by proximal threats and anxiety/anxiety responses evoked by distal and uncertain threats (Grillon, 2008, Perusini and Fanselow, 2015, Mobbs et al., 2020) (although this may be an oversimplification at the neurobiological level (Fox and Shackman, 2019, Perusini and Fanselow, 2015, Daniel-Watanabe and Fletcher, 2021). Critically, while fear, anxiety and the responses they elicit (such as avoidance) can be adaptive, their excessive and/or inappropriate expression can have severe negative impacts on daily functioning. This, in conjunction with an individual’s set of beliefs and attitudes (Beck and Clark, 1988), may lead to fear- and anxiety-related disorders such as phobias or generalised anxiety disorder, respectively (Kessler et al., 2005) which collectively constitute leading causes of disability (Vos et al., 2017).

The computational approach, which we define here as inference of the unobserved causes of behaviour through **generative models** (Stephan and Mathys, 2014) (see Box 1 for the Glossary of bolded terms), has seen a surge in interest in the study of fear and anxiety (Bach and Dayan, 2017). This approach aims to derive the computations underlying defensive behaviours, as well as how these computations may be implemented at the neural level. This focus on computation aims to move the field beyond simply describing symptoms and towards understanding the cognitive and neurobiological *mechanisms* underlying fear and anxiety behaviours. Moreover, computational modelling of behaviour may offer superior measurement properties compared to more traditional summary-statistic-based methods (Price et al., 2019, Tipples, 2015). In this mini-review, we discuss recent themes emerging from this field, namely the roles of a) learning; b) approach-avoidance conflict; and c) uncertainty in human fear and anxiety.

Box 1 **Glossary.**

*Computational concepts*.

**Active inference** (Friston et al., 2017)**.** Active inference is a Bayesian framework which assumes perception and action as problems of inference (Friston et al., 2013). Here, perception is defined as inferring the latent states of the world that cause observable outcomes. Action is defined as inferring policies (sequences of actions) that must be adopted to obtain certain outcomes. These problems are separated by assuming that actions are consequent on the predictions formed by perception. Like model-based reinforcement learning, the generative model includes information about future states and policies, which can be used to discern the optimal actions to obtain favourable outcomes. Optimising behaviour is achieved by maximising the observable evidence for this generative model (via the minimisation of variational free energy).

**The drift**-**diffusion model** (Ratcliff, 1978)**.** The drift-diffusion model is a generative model which integrates choice and response time data. In the simplest form, it describes the accumulation of evidence (perceptual or value-based) for two competing responses (for example, whether a stimulus is large or small, or whether one stimulus is more valuable than the other). Evidence is accumulated until a threshold is reached, indicating more evidence for one response over the other. The form of evidence accumulated dictates choice, while the rate of evidence accumulation affects response times (faster evidence accumulation leads to shorter response times, and vice versa). The basic model has four parameters: a drift rate (controlling evidence accumulation); a threshold boundary (controlling how much evidence is required for a decision); initial evidence (for a priori biases in evidence); and a non-decision time (for decision-irrelevant factors preceding decisions).

**Generative models.** Mathematical models which describe how certain data (for example behaviour, response times, neural responses, or a combination of these data) are ‘generated'. With respect to generative modelling of behaviour, this consists of building models which represent a researcher’s hypotheses about the unobserved causes (e.g. computations that the brain performed) of the observed behavioural data. These models typically include free parameters which can reflect individual differences in cognitive processes (for example, a learning rate). The term ‘generative’ also implies that one can generate trial-by-trial artificial data from the model, with which to compare to the original data (often referred to as ‘posterior predictive checking’ (Gelman and Hill, 2007)) to determine how well the model captures the data. Examples include reinforcement learning models, logistic choice models, and drift-diffusion models.

**Prospect theory** (Kahneman and Tversky, 1979)**.** Prospect theory is a generative model of risky economical decision-making. The model describes how to translate some monetary value (say the potential to earn £100) and its associated probability of occurrence (say 50% likelihood of earning the £100) into individual-specific subjective values. Biases in decision-making such as loss aversion can be modelled by transformations (e.g. scaling) of the value, which can also account for individual differences in sensitivity to value. Similar transformations can be performed for probabilities to model risk aversion.

**Reinforcement learning.** Reinforcement learning provides a framework for modelling reward-driven (or indeed punishment-driven) behaviour. In the context of modelling human or animal behaviour, a generative model takes the form of an ‘agent’ that performs certain actions within a certain environment, where the agent attempts to accrue as much reward as possible whilst minimising losses/punishment. Different agents can be specified to rely on different behavioural strategies (or ‘policies’). In the field of artificial intelligence, these strategies are commonly categorised into model-free and model-based (see below).

**Reinforcement learning; model-free reinforcement learning.** Model-free learning entails learning, from experience and trial-and-error, what actions are favourable (i.e. produce reward/avoid punishment, or otherwise lead to other favourable states) given the current state of the environment. This is a computationally efficient form of behavioural control since a model-free agent only needs to rely on state-action values to perform favourable actions. Put another way, a model-free agent does not require an explicit causal understanding of which actions lead to certain outcomes/other states (unlike model-based agents, see below).

**Reinforcement learning; model-based reinforcement learning.** Model-based strategies rely on an understanding of the causal structure of the environment, or more specifically an understanding of the most likely outcome given the current state and candidate action. Therefore, this causal structure can be used to plan future actions and consequent outcomes. Compared to model-free learning (see above), model-based planning is potentially computationally costly (scaling with the complexity of the environment) but confers greater behavioural flexibility, especially in dynamic environments.

**State** values. Information about an agent’s predictions about what positive or negative outcomes might occur in a certain ‘state’ of the environment is encoded in the state value. For example, this could be the agent’s prediction of an electric shock occurring when the agent observes a certain stimulus. In single-step reinforcement learning, this represents the predicted likelihood or magnitude of reward or threat associated with the state, whereas in multi-step reinforcement learning, it also includes future rewards/threats that might occur given the occurrence of the state. Typically, positively valenced outcomes (i.e. things one wants to obtain/approach such as food) lead to positive state values, whilst negatively valenced outcomes (i.e. things to be avoided such as pain) lead to negative values.

**State-action** values. In a similar fashion to state values, state-action values encode an agent’s predictions about what outcome might occur, given the state of the environment and the agent’s action. For example, this could be the agent’s prediction of an electric shock occurring if the agent presses a button (say rather than not pressing the button) in response to a certain stimulus.

*Forms of anxiety - across this mini-review, we have used a number of terms that refer to different forms of anxiety. For clarity, we provide a brief description of the general terms used.*

**Pathologically anxious** individuals. We use this phrase to denote individuals who report clinically relevant symptoms of anxiety, for example as assessed by clinical interviews.

**Somatic vs cognitive** anxiety. Previous research has proposed a distinction between these two dimensions of anxiety (Koksal and Power, 1990, Ree et al., 2008). Somatic anxiety involves physical symptoms of anxiety, such as hyperventilation, sweating and muscle tension. On the other hand, cognitive anxiety involves symptoms relating to thoughts, such as worry and difficulty concentrating.

**State** anxiety. The transient experience of anxiety, which may be induced by experimental procedures (e.g. anxiogenic tasks/manipulations) or experienced naturally.

**Trait** anxiety. Anxiety symptoms and related thoughts which are experienced over a relatively long period by an individual, which can sometimes be considered as part of their stable characteristics or personality.

## 2 Aversive learning

Learning to predict threat from the environment and act accordingly to avoid it is of fundamental importance for the survival of organisms. Building on the seminal work of Rescorla-Wagner (Rescorla and Wagner, 1971) and Pearce-Hall (Pearce and Hall, 1980), **reinforcement learning** (Sutton and Barto, 2018) (see Box 1) represents one of the most popular frameworks for quantitatively modelling learning about threats and punishments. It can be broadly divided into Pavlovian fear conditioning and instrumental learning (for a comprehensive classification of threat learning/behaviour, we direct the reader to this recent review (LeDoux and Pine, 2016)). In this section, we describe various reinforcement learning strategies that humans employ when learning to predict or avoid threat, and how these relate to symptoms of fear and anxiety.

### 2.1 Fear conditioning

**Fig. 1:**
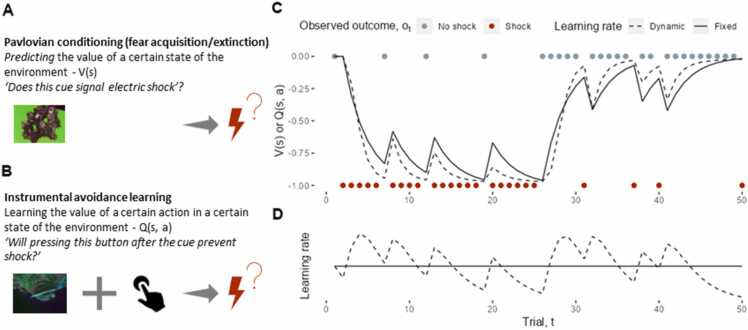
Pavlovian fear conditioning and instrumental avoidance learning. A) In generative models of Pavlovian fear conditioning, agents update an expectation of the value, $V\left(s\right)$, of a certain state of the environment, s. In a simple fear conditioning experiment, this could be the expectation of a shock given the presentation of a certain cue. B) In instrumental learning, agents instead learn the expected value, $Q\left(s,a\right)$, of an action, a, given state, s. This could be where a participant learns to press a button after seeing a certain cue to avoid shock. C) Conceptual data in a common reversal learning paradigm (which involves elements of both value acquisition and extinction), where participants learn to associate a cue (Pavlovian conditioning) or a button-press after observing a cue (instrumental learning) with shock or no shock. The lines represent how the expected values, $V\left(s\right)$ and $Q\left(s,a\right)$, change after each observed outcome (red and grey dots represent shock and no shock outcomes on each trial, respectively). Initially in the task, the cue/cue and button-press leads to shock with 80% probability, but in the second half of the task, they only lead to shock on 20% of trials. For demonstration, we force the agent to button-press on each trial, but in practice, participants can choose between multiple actions. The dynamics of $V\left(s\right)$ and $Q\left(s,a\right)$ can be modelled through similar mechanisms such as the Rescorla-Wagner learning rule. We demonstrate two models: a simple Rescorla-Wager model which involves a fixed learning rate (in solid lines), and a ‘hybrid Rescorla-Wagner Pearce-Hall’ model which allows for a dynamic learning rate (In dashed lines). In both models, the agents learn that the cue/cue and button-press is initially associated with the shock (as the expected values become negative). When the contingencies change midway through the task, the agents learn that they are no longer associated (i.e. the expected values become more positive). D) Trial-level learning rates in each model. The fixed learning rate stays constant. The hybrid model relies on a high learning rate at the start of the task and midway though (when the contingencies change), which decreases after these moments. The high learning rate captures the ‘unpredictability’ of recent outcomes, as the agent has not yet learned the current contingency. The learning rate decreases as the outcomes become more predictable. The hybrid model is typically a better explanation of human behavioural data (Li et al., 2011, Homan et al., 2019, Tzovara et al., 2018, Piray et al., 2019). For this simulation, we used an initial learning rate of 0.3 for both models, and a second-order learning rate (see η in the main text) of 0.3 in the hybrid model.

Pavlovian fear conditioning was one of the earliest experimental models of fear and anxiety (Watson and Rayner, 1920), and is thought to describe the acquisition of fear in psychopathology. Briefly, Pavlovian conditioning involves the development of a learned reflexive response to a neutral cue, when paired with a biologically significant (e.g. threatening) stimulus/event. The Rescorla-Wagner learning model provides an elegant explanation of fear conditioning, by ascribing values (**state values**; see Box 1), $V\left(s\right)$, to certain states of the environment, s, such as the presence of a cue predicting danger, at time, t (Fig. 1). Agents learn from discrepancies between their expected outcome and the actual outcome, $o_{t}$ (e.g. an electric shock which can be represented as a value of −1 [or sometimes 1 if there are no rewards present in the task, see Box 1]), which results in a prediction error, ${\mathrm{PE}}_{t}=o_{t}-V_{t}\left(s_{t}\right)$. This prediction error is used to update the state value via $V_{t+1}(s_{t})=V_{t}(s_{t})+\alpha \cdot PE_{t}$, where learning is scaled by the learning rate, $\alpha \in \left[0,1\right]$. Negative $V\left(s\right)$ values signal that the agent expects an aversive outcome to occur given s, modelling fear. The neurobiological validity of the Rescorla-Wagner learning model is supported by neural correlates of prediction errors during fear conditioning tasks in the human striatum (Delgado et al., 2008, Robinson et al., 2013, Seymour et al., 2007), with similar models (McNally et al., 2011) and neural correlates (McNally and Westbrook, 2006, Stanley et al., 2021) observed in the non-human animal literature. Further, autonomic measures (e.g. skin conductance) track model-predicted changes in $V\left(s\right)$ (Li et al., 2011, Zhang et al., 2016) supporting the notion of state-values as signalling fear.

The classical Rescorla-Wagner model assumes a static learning rate, but models that allow for dynamic learning rates commonly provide more parsimonious accounts of fear conditioning (Li et al., 2011, Homan et al., 2019, Tzovara et al., 2018, Piray et al., 2019) (Fig. 1 C-D). Inspired by the Pearce-Hall learning model (Pearce and Hall, 1980), these models (which are referred to as ‘hybrid’ models as they combine Rescorla-Wagner and Pearce-Hall mechanisms) incorporate the notion of ‘predictability’ into the learning process, by scaling the rate of learning in proportion to the magnitude of recent prediction errors: $\alpha_{t+1}\left(s_{t}\right)=\eta \cdot \left|{\mathrm{PE}}_{t}\right|+(1-\eta )\cdot \alpha_{t}\left(s_{t}\right)$, where the rate of scaling is parameterised by $\eta \in \left[0,1\right]$. This means that greater error magnitudes (i.e. low predictability) call for rapid learning, whereas learning is slower for low error magnitudes (i.e. high predictability). Neural correlates of model-predicted changes in learning rate in the amygdala (Li et al., 2011, Zhang et al., 2016) and dorsal anterior cingulate cortex (Piray et al., 2019) provide support for the potential neural implementation of this computational mechanism. These effects parallel findings from the animal literature, where for example amygdala inactivation leads to behaviour consistent with learning rate impairments (Roesch et al., 2010). With respect to pathological fear, PTSD symptoms in combat-exposed veterans were positively associated with learning rate variability (Homan et al., 2019) (i.e. greater values of η), meaning that those with more severe symptoms were more sensitive to changes in the predictability of outcomes. The opposite effect was reported, however, for trait social anxiety (Piray et al., 2019), perhaps indicating unique learning mechanisms across these disorders (see below for a further discussion of anxiety, learning rate and uncertainty). Finally, recent work has also shown that visual attention modulates the rate of learning, by selectively strengthening $V\left(s\right)$ for attended cues (Wise et al., 2019).

Fear conditioning can also describe how fear is overcome – a process referred to as *fear extinction*. During extinction, a cue previously associated with threat is repeatedly presented in its absence, which leads to decreases in cue-induced fear responses (Bouton, 1993). This is utilised clinically in exposure therapy (Craske et al., 2006, Hofmann, 2008), which aims to reduce pathological fear via extinction. Unfortunately, fear sometimes spontaneously re-emerges even after extinction learning, which is difficult to reconcile with Rescorla-Wagner accounts of learning and presents a hurdle for therapy. The current theoretical understanding of this effect is that extinction leads to a new ‘safety’ association which competes with the original association, potentially causing recovery of fear (Bouton et al., 2021). However, recent studies suggest that this might be subject to individual differences in learning about latent causes of the environment. Indeed, a greater tendency to form new safety associations predicted later recovery of fear in humans, whereas those who were more likely to modify original fear associations were less likely to re-experience fear (Gershman and Hartley, 2015). Convergent evidence from a rodent study also indicates that gradual extinction learning, compared to abrupt changes in outcome contingencies, promoted modification of the previous association over the formation of a new association, and reduced the likelihood of later fear (Gershman et al., 2013). Perhaps counterintuitively, re-experiencing symptoms in PTSD (which can be considered a clinical presentation of recovery of fear) were associated with a *reduced* tendency to form new safety associations (Norbury et al., 2021). This implies that although individual differences in latent cause learning are relevant to pathological fear, it is not a simple unidirectional relationship and other factors are likely involved (such as biases for *which* associations are evoked if multiple associations are stored).

### 2.2 Instrumental/avoidance learning

Instrumental learning tasks, in which an agent’s actions determine outcomes, can be used to model avoidance behaviours. These tasks can encompass both *active* avoidance, in other words situations where certain actions can avert aversive outcomes, and *passive* avoidance, where *inaction* avoids aversive outcomes (or conversely where actions may lead to aversive outcomes). In both cases, learning **state-action values** (see Box 1), $Q\left(s,a\right)$, under certain states, s, and following certain actions, a, can be modelled through **model-free reinforcement learning** (see Box 1), which relies on an error-dependent learning mechanism similar to that in fear conditioning: $Q_{t+1}\left(s_{t},a_{t}\right)=Q_{t}\left(s_{t},a_{t}\right)+\alpha \cdot (o_{t}-Q_{t}\left(s_{t},a_{t}\right))$ (Fig. 1; note close variants of model-free RL to that presented here which tie actions to cumulative expected reward can solve 'multistep' problems). This provides an account of how humans learn to act to avoid outcomes such as pain (Jepma et al., 2022, Eldar et al., 2016), with corresponding neural correlates of instrumental prediction errors in the striatum (Eldar et al., 2016) and periaqueductal gray (Roy et al., 2014). Further, when these models are extended to account for asymmetries in learning from safety and threat (the omission or occurrence of an aversive outcome, respectively), by implementing separate learning rate parameters for safety and danger, $\alpha_{\mathrm{safety}}$ and $\alpha_{\mathrm{threat}}$, subtle differences can be found across different forms of anxiety on aversive learning performance. In one study (Wise and Dolan, 2020), a double dissociation emerged across trait **cognitive** vs **somatic anxiety** (see Box 1), where cognitive anxiety was associated with a bias for learning from threat, whilst the opposite was true for somatic anxiety and also trait compulsivity.

**Fig. 2:**
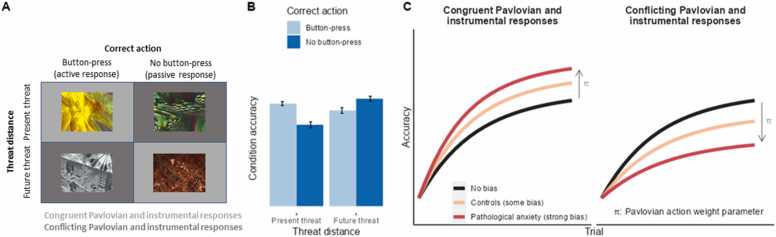
The reinforcement learning task of Millner et al. (2018). A) Task structure. Participants learnt to press a button (active response) or not (passive response) in order to avoid already-present or future threat. For the present threat condition, a continuous aversive sound was played until the correct action was made, which probabilistically terminated the sound. For the future threat condition, the trial began in silence and the correct action could probabilistically prevent the sound from being played (incorrect actions would instead result in the sound being played). Threat distance (present/future) was crossed with correct action (button-press/no button-press) to produce four conditions in the task. B) Illustration of data from Millner et al. (2018). Mean condition accuracy and standard errors extracted from the original paper. Participants were better at learning to make passive rather than active responses for future threat, but the opposite was true for already-present threat. C) A conceptual demonstration of the effect of Pavlovian response biases during instrumental avoidance learning. The curves reflect improved accuracy over trials as individuals learn the correct actions in each condition. If the correct (instrumental) action is congruent with Pavlovian responses, namely to emit active responses to avoid present threat and passive responses to avoid future threat, learning performance is augmented in healthy controls (orange line) and even further in pathological anxiety (red line). However, if the correct action conflicts with these Pavlovian response biases, learning performance is hindered and this effect is again greater in pathological anxiety. This can be captured by a Pavlovian action weight parameter, π - learning in individuals with pathological anxiety (and depression) is characterised by greater values of π (Mkrtchian et al., 2017).

State values can provide a computationally efficient mechanism of instrumental responding, by biasing approach-relevant responses under positive $V\left(s\right)$ (i.e. when states predict positive outcomes) and avoidance-relevant responses under negative $V\left(s\right)$ in a Pavlovian manner (an effect referred to as Pavlovian-instrumental transfer (Dickinson and Balleine, 1994)). This is implemented by adding an action-weight to action-outcome associations, such that the overall value for the action is given by $Q_{t}\left(s_{t},a_{t}\right)+\pi \cdot V_{t}\left(s_{t}\right)$. Here, π parameterises an agent’s tendency to behave according to Pavlovian mechanisms (Fig. 2). This can constitute an efficient mechanism of evading threats as a state value, which comprises two components (the state and its expected value), is sufficient to produce defensive behaviour. In contrast, instrumental learning requires a further ‘action’ component to build the state-action-value association. Humans readily learn state-action-value associations, in other words to emit or omit certain actions to avoid punishment (Guitart-Masip et al., 2012; Millner et al., 2018). At the same time, this learning is also biased by state values, which inhibit motor responses when learning to avoid future punishment in a manner consistent with disengagement/freezing (Guitart-Masip et al., 2012, Roelofs, 2017), but promote active escape behaviour when a threat is already present (Millner et al., 2018) (Fig. 2). These effects parallel classical learning accounts which posit that threat makes some actions more likely to be emitted (e.g. freezing) than others (Bolles, 1970, Hershberger, 1986). Finally, the disadvantage of Pavlovian mechanisms is that response biases can be challenging to overcome when Pavlovian and instrumental mechanisms conflict – for example, when one must make an active response in the face of potential threats (rather than freezing) leading to suboptimal behaviour in such situations (Guitart-Masip et al., 2012, Millner, 2018) (Fig. 2). This effect is exacerbated in **pathologically anxious individuals** (see Box 1), especially under acute anxiety induced by threat-of-shock (Mkrtchian et al., 2017), and this is thought to underlie the excessive avoidance behaviours observed in pathological anxiety.

The behaviours discussed so far are typically considered ‘single-step’ problems, where an agent is only required to learn the appropriate response to perform in a single state of the environment (e.g. how to respond to one particular cue). However, most real-world scenarios are more likely to be *multi-step*, where an agent must perform a sequence of actions across multiple states of the environment to obtain a particular outcome. In keeping with a distinction of fear and anxiety as relating to proximal/distal threats, single-step problems might provide better models of fear as the threat is potentially associated with the present state of the environment, whereas anxiety might be more associated with multi-step learning, where the potential threat is diffused across the multiple states and actions in the environment. How humans solve multi-step aversive learning is described through **model-based reinforcement learning** (see Box 1), which relies on a ‘model of the world’, in other words an understanding of the different states of the environment and the transitions across states, to infer optimal behaviour.

**Fig. 3:**
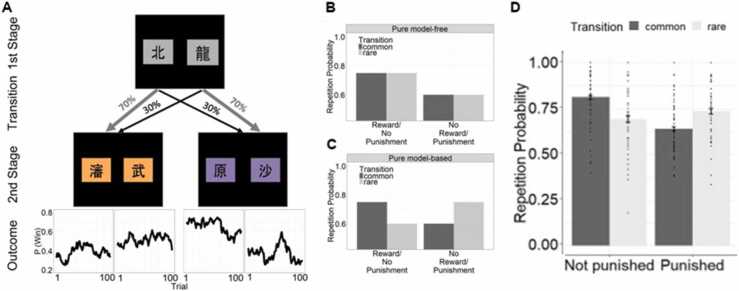
The two-step task of Sebold et al. (2019). A) Task structure. On each trial, participants made a series of binary choices. The first-step choice (grey cues) probabilistically leads to distinct second-stage states (orange or purple cues), with one choice leading to one second-stage state, for example the orange set, on 70% of trials (referred to as ‘common’ transitions; light grey arrows), and the other second-stage state, for example the purple set, on the remaining 30% of trials (‘rare’ transitions; black arrows). The opposite was true for the other first-stage choice. At the second stage, the second-step choice produced an outcome that depended on choice-specific outcome probabilities that varied over time. The outcome is depicted here as ‘Win’ for reward, but it is possible to also have punishments as the outcomes. B-C) Model-free and model-based reinforcement learning strategies entail different probabilities of repeating the first-stage choice of the previous trial, given the outcome on the previous trial. B) The model-free strategy does not take into account the transition structure of the task and tends to simply repeat first-stage choices if on the previous trial, that choice produced a reward or avoided a punishment. This manifests as a main effect of previous reward/punishment on first-stage choice repetition. C) The model-based strategy utilises the structure of the task and accounts for whether the previous trial involved a common or rare transition, leading to an interaction effect of previous reward/punishment and transition (common/rare) on first-stage choice repetition. D) Illustration of data from Sebold et al. (2019). Mean and error bars for repetition probabilities by condition. Dots represent individual participant data. When learning to avoid punishments in this task, participants typically show a mixture of model-free and model-based influences on choice (i.e. both a main effect of previous outcome and an interaction effect of transition type and previous outcome on choice repetition probability). The figures are reproduced from Sebold et al. (2019), which were published under CC BY 4.0.

The dominant paradigm for measuring model-free and model-based learning strategies to date has been the two-step task (Daw et al., 2011) (Fig. 3), which involves a series of two actions to obtain a certain outcome. Model-free and model-based strategies imply separate predictions for the first action in the sequence, and thus individual choices here indicate a reliance on one strategy over the other. Humans tend to use both model-free and model-based learning strategies for threat avoidance (Sebold et al., 2019, Wang et al., 2018) (Fig. 3), as is the case with reward learning (Daw et al., 2011). The evidence relating model-based planning to anxiety is mixed: on one hand, some studies have reported that individuals favour model-free strategies over model-based in aversive environments (when viewing aversive images) (Sebold et al., 2019) and under social stress (Park et al., 2017). Yet, experimentally-induced (hypercapnic gas) and naturalistic anxiety (panic attacks/life stress) do not appear to impact model-based learning (Gillan et al., 2021) . Further research is needed to determine whether these discrepancies in findings are due to methodological differences or false positives. Instead, the evidence suggests that trait compulsivity, rather than **trait anxiety** (see Box 1), is associated with decreased reliance on model-based learning (Gillan et al., 2021, Gillan et al., 2016) – an effect which has been proposed to be driven by impairments in state-transition learning, in other words that compulsive individuals have difficulty learning the relationships between states of the environment (Sharp et al., 2021), which leads to reduced confidence in one’s ability to navigate these states.

Arguably, the two-step task may not be sufficiently complex to reveal impairments of planning in anxiety. Findings from a recent study (Sharp et al., 2022) implementing a multigoal pursuit task suggest that more naturalistic planning problems (balancing multiple goals) may be more sensitive to anxiety-related effects. In the task, healthy individuals learned the likelihoods of observing two tokens for each of two possible actions: one token was associated with monetary reward, and the other with monetary punishment. However, individuals were instructed on each trial that only one token would be relevant on each trial (i.e. on reward trials, obtaining the reward token could lead to a reward, while obtaining the punishment token had no consequence, and vice versa for punishment trials). Thus, the task required individuals to use only goal-relevant information in their choices. In the task, individuals struggled to disengage from punishment-relevant information on reward trials, and this effect was positively correlated with chronic worry, a central feature of pathological anxiety (American Psychiatric Association, 2013). This effect suggests an impairment in model-based planning, where individuals with more severe worry appear to plan for threat avoidance even in explicitly safe environments – a finding which parallels ideas from clinical theories of worry which posit that worry constitutes maladaptive planning to avoid imagined/distal threat (Mathews, 1990). Future work will be required to determine the generality of planning impairments to trait anxiety, or whether the effect is specific to forms of anxiety that involve higher cognitive functions (such as worry), compared to those that do not (such as somatic anxiety) (Sharp et al., 2015).

In brief, humans rely on multiple computational strategies to predict and avoid threat. There is strong evidence that fear is acquired and extinguished based on error-driven learning, and individual differences in beliefs about the latent state of the environment might predict failure to extinguish fear. Pavlovian and instrumental processes can also interact in driving behaviour, and this appears to be exacerbated by pathological anxiety. Anxiety, specifically, may be associated with differences in model-based planning, especially in worry. Similarly, different psychiatric symptoms (e.g. social anxiety vs PTSD, cognitive vs somatic anxiety) show subtly different learning characteristics, such as biases in learning selectively from safety or threat.

## 3 Approach-avoidance conflict

Real-world decisions are rarely reducible to evasion of a single threat; instead, the consequences of a single action often simultaneously involve positive *and* negative outcomes. This means that one needs to decide to pursue reward at risk of incurring a punishment, or avoid that punishment and forsake the potential reward. Such situations, which are referred to as involving *approach-avoidance conflict*, are exploited in animal models of anxiety such as the Vogel conflict test (Vogel et al., 1971), where the drive to approach reward (e.g. to obtain water) is pitted against the drive to avoid threat (e.g. to avoid an electrical shock). Excessive or consistent avoidance in situations of approach-avoidance conflict entails giving up reward in order to avoid potential threat, which reflects maladaptive avoidance in pathological fear/anxiety (Aupperle and Paulus, 2010, Loijen et al., 2020) where important things in life may be sacrificed in order to avoid stressors/threat. Here, we discuss some initial efforts to explore approach-avoidance conflict paradigms through a computational lens.

**Fig. 4:**
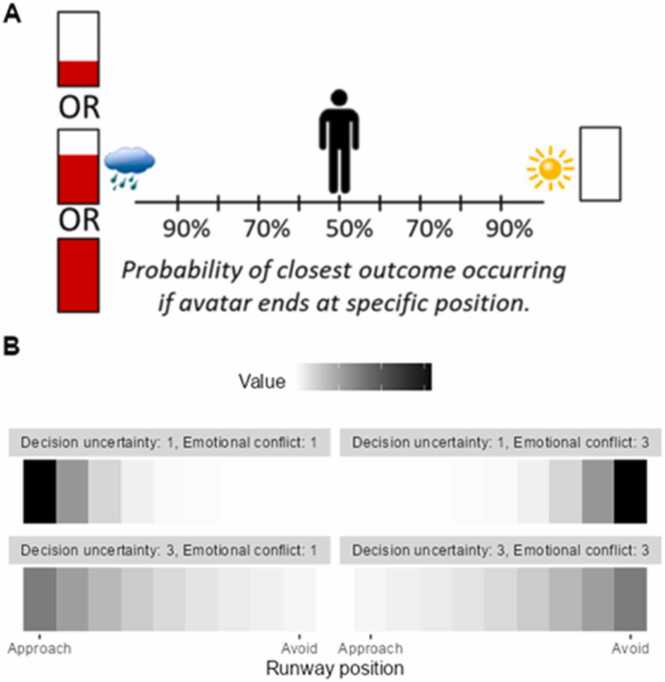
The approach-avoidance conflict task of Smith et al. (2021). A) Task structure. Participants decided on their preference for viewing an aversive image and hearing an aversive sound (represented by the raincloud) in return for varying levels of monetary reward (represented by the proportion of a bar filled in red), OR viewing a pleasant image and hearing a pleasant sound (represented by the sun) but with no monetary reward (see the empty bar). Responses were made by moving an avatar left or right on a runway which corresponds to probabilities that participants will see either the left or right outcomes, reflecting their relative preference. B) A conceptual demonstration of the effect of the key parameters of the generative model. The aversive stimuli and reward outcome is referred to as the ‘Approach’ outcome, and the pleasant stimuli with no reward outcome is referred to as the ‘Avoid’ outcome. The subjective value of each position of the runway is represented in greyscale, with darker tones representing greater value. Subjective value depends on two parameters. Firstly, the emotional conflict parameter captures the expected aversiveness of the aversive stimuli relative to the monetary reward, with higher emotional conflict indicating greater aversiveness. Low emotional conflict (top-left, bottom-left) means that individuals value the ‘Approach’ outcome more than the ‘Avoid’ outcome because the expected aversiveness of the aversive stimuli is low – this reverses as emotional conflict increases (top-right, bottom-right). Secondly, the decision uncertainty parameter reflects how confident participants are in their choices. High decision uncertainty (bottom-left, bottom-right) indicates lower confidence in discerning the value of each runway position, leading to a broader distribution of value across the runway. Conversely, low decision uncertainty (top-left, top-right), or greater confidence, leads to a narrower distribution of value. Fig. A is reproduced from McDermott et al. (2021), which was published under CC BY 4.0.

Recent studies have attempted to adapt rodent conflict tests for humans, typically in tasks where individuals decide on whether to accept or reject an offer of some aversive outcome (e.g. an electric shock) alongside some monetary reward (Aupperle et al., 2011, Ironside et al., 2020) (Fig. 4 A). Using an **active inference** model (Friston et al., 2013, Friston et al., 2017) (see Box 1), a Bayesian generative model of decision-making, one study compared the roles of ‘emotional conflict’,^1^ defined as the relative value of the aversive outcome compared to the reward, and ‘decision uncertainty’, defined as the difficulty in making the decision (Smith et al., 2021) (Fig. 4B). In keeping with the notion of the task as measuring anxiety-related behaviour, **state anxiety** during the task was correlated with greater emotional conflict, meaning that those who found the aversive outcomes more unpleasant were more anxious during the task. However, this effect did not extend to group differences between pathologically anxious and depressed individuals and non-symptomatic controls. Interestingly, the symptomatic group did show greater decision uncertainty, relative to controls, perhaps reflecting findings from clinical psychology that anxiety symptoms, specifically chronic worry, are associated with indecision (Snyder et al., 2014).

Approach-avoidance conflict has also been modelled in tasks simulating foraging under predation, where individuals collect rewards at risk of being caught by a predator (Bach, 2021). Avoidance behaviour in these tasks scales with threat probability (Bach, 2015, Loh et al., 2017, Qi et al., 2018), with greater threat probability leading to greater avoidance. Even when individuals make approach responses, response latencies are greater under high vs. low threat probabilities, demonstrating that behavioural inhibition occurs even during approach behaviour, and this effect positively correlates with trait anxiety (Bach, 2015). Neuroimaging studies using these tasks have repeatedly implicated the ventral hippocampus (Ito and Lee, 2016, O'Neil et al., 2015, Bach et al., 2014), consistent with previous reports of its involvement in anxiety/fear and reward circuitry (Shin and Liberzon, 2010, Russo and Nestler, 2013), but its precise computational role has yet to be established.

Lastly, asymmetries in reward and aversive learning have been studied in instrumental learning paradigms where individuals learn to simultaneously pursue reward and avoid punishment, specifically where on each trial, an action could potentially lead to the delivery of both the reward and the punishment. Compared to healthy controls, the learning performance of pathologically anxious and depressed individuals was characterised by higher punishment learning rates (Aylward et al., 2019), meaning that they relied on fewer observations to estimate the aversive value of a certain action, but there was no group difference for the reward value of actions. The group difference in punishment learning rates was corroborated by a recent meta-analysis of 27 reinforcement learning studies across more than 3000 participants (Pike and Robinson, 2022). This study used a novel simulation-based approach that allowed for pathologically anxious/depressed individuals to be compared with controls across a range of different learning tasks, and by using simulations of their behaviour, the study found that the symptomatic group showed not only higher punishment learning rates, but also lower reward learning rates, compared to controls. This implies that anxious/depressed individuals show negative biases when learning about rewards and punishments, learning slower about the former and faster about the latter. Of note, however, this analysis pooled across anxiety and depression studies, leaving open the question of the effect’s specificity to anxiety.

In sum, despite the reliance of many animal models of fear and anxiety on approach-avoidance conflict (Campos et al., 2013), there is surprisingly limited work applying computational approaches to its understanding. Initial findings indicate roles of uncertainty in making decisions under conflict and asymmetries in learning about rewards and punishments in pathological anxiety (and depression), but more work will be required to better understand how individuals trade off rewards and punishments, perhaps by better use of translational paradigms (Kirlic et al., 2017).

## 4 Decision-making under uncertainty

Central to the definition of anxiety is the notion of *uncertainty* of threat. Pathologically anxious individuals often report negative beliefs about uncertainty and its implications, a concept referred to as *intolerance of uncertainty (*Dugas et al., 1997*)* and this is considered by some to be a core component of certain anxiety disorders (Dugas et al., 1998, Carleton et al., 2012). Indeed, multiple cognitive mechanisms may contribute to maladaptive responses to uncertainty in pathological anxiety (Grupe and Nitschke, 2013). In the next section, we discuss negative biases in decision-making in the face of uncertainty in anxiety.

### 4.1 Negative biases for uncertain outcomes

**Fig. 5:**
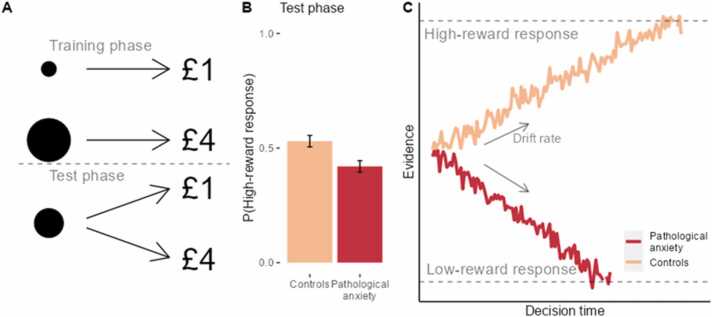
The perceptual decision-making task of Aylward et al. (2020). A) Task structure. Participants were first trained to discriminate between two stimuli (here, a small and large circle) using two response keys. Pressing the correct response key for one of the stimuli deterministically leads to a high reward, whereas the correct response key for the other stimulus leads to a smaller reward (response-reward associations were counter-balanced across participants). In the test phase, an intermediate-sized stimulus was presented, for which both response keys lead to their respective rewards (high reward for the high reward response, low reward for the low reward response) with equal probability. B) Illustration of data from Aylward et al. (2020). Mean proportion of high-reward responses to the intermediate stimulus by group (pathologically anxious vs healthy controls) and standard errors extracted from the original paper. Pathologically anxious individuals were less likely to respond to the ambiguous stimulus with the high-reward response, and healthy controls showed the opposite effect. C) This effect was computationally captured in a drift-diffusion model, specifically through the drift rate parameter which controls the rate and direction by which evidence is accumulated (i.e. for the high-reward vs the low-reward response). In the study, pathologically anxious individuals (red line) showed more negative drift rates, compared to the controls (orange line).

A key finding from early cognitive research was that anxious individuals demonstrate a negative interpretational bias (Beck and Clark, 1988, Beck and Clark, 1997, Hirsch et al., 2016), that is, a tendency to interpret ambiguous information in a negative light. A computational basis for this has been proposed in a perceptual task (Aylward et al., 2020) translated from an animal model of negative bias (Harding et al., 2004, Hales et al., 2016). Pathologically anxious and depressed individuals were more likely to interpret ambiguous stimuli as if they would lead to the worst of two possible outcomes (Fig. 5), relative to healthy controls. **Drift**-**diffusion modelling** (see Box 1) revealed that this bias was driven by group differences in drift rates; in other words, the symptomatic group was more likely to accumulate negative evidence about the ambiguous stimulus during deliberation. This effect parallels findings from lexical decision-making (White et al., 2010) and facial discrimination tasks (Glasgow et al., 2022), which together suggest that pathologically anxious individuals tend to build more negative representations of the environment, which may lead to maladaptive behaviour especially under uncertainty.

What might drive negative biases under uncertainty? A plausible explanation is that anxiety is associated with a prior belief that negative events occur more frequently than positive events (Butler and Mathews, 1983). A signal detection theory analysis of the perceptual task described above (Aylward et al., 2020) is consistent with negatively biased prior beliefs about outcome probabilities (Locke and Robinson, 2021). Further, modelling individual risk sensitivity using **prospect theory** (Kahneman and Tversky, 1979) (see Box 1) showed that anxious individuals are more sensitive to risk in economical decision-making compared to healthy controls, leading to preferences for smaller yet guaranteed (i.e. more certain) rewards over gambles for greater rewards (Charpentier et al., 2017), which can be interpreted as over-weighting of the probability of disadvantageous outcomes. Relatedly, recently proposed computational models of anxiety (Zorowitz et al., 2020) and obsessive-compulsive disorder (Fradkin et al., 2020) show through simulations that maladaptive avoidance behaviour can stem from pessimistic beliefs about personal ability to avoid threat in the future (which is intrinsically uncertain), leading individuals to make inappropriate/excessive avoidance responses in the present.

### 4.2 Stochasticity vs volatility

Another line of research has focused on dissecting uncertainty into two components: stochasticity and volatility (Piray and Daw, 2021). Stochasticity arises when learning the value of a state or state-action pair if an agent observes variance in the outcomes (i.e. if the relationships are non-deterministic, as much the work in the previous section involved), leading to uncertainty in the precision of the value estimate. On the other hand, volatility involves changes in the state or state-action values themselves, which leads to uncertainty in the estimate of the value. Stochasticity and volatility have diverging consequences for efficient learning: high volatility, in other words when there is a lot of change in the values of states/state-action pairs, requires agents to update their values estimates more frequently, which can be achieved by increasing one’s learning rate, α. High stochasticity, or where there is high variance in the observed outcomes (assuming the value is stable) calls for slower learning and lower α, as each individual outcome is less informative about the true value. Humans can adaptively adjust their learning rates to variable stochasticity (Lee et al., 2020) and volatility (Behrens et al., 2007).

The previous section of this review showed that anxiety is associated with negative biases when outcomes are stochastic. There is also evidence associating anxiety with impaired learning in volatile environments. Specifically, when learning to avoid threat under conditions of low and high volatility (with stable stochasticity across conditions), individuals with low trait anxiety adjusted their learning rates across conditions according to the optimal strategy, but high trait anxiety was associated with less flexibility in learning rates, leading to sub-optimal performance (Browning et al., 2015). Similar effects have also been found during reward learning (Huang et al., 2017, Gagne et al., 2020) and social decision-making (Lamba et al., 2020), indicating a domain-general impairment in adapting to volatility. Obsessive-compulsive and fear symptoms have also been associated with impairments in state-transition learning under low and high volatility, specifically with sub-optimally fast learning in stable conditions and slow learning in volatile conditions (Sharp et al., 2021), indicating a transdiagnostic role of learning under volatility in threat-related psychopathology. Towards a neural mechanism of learning rate inflexibility, one study found that the dorsal anterior cingulate cortex in healthy controls tracks changes in learning rate over trials, but this was not the case for anxious individuals (Piray et al., 2019). Finally, a recent simulation-based approach suggests that learning rate inflexibility can be explained if an agent is biased to perceived stochasticity as low and constant but volatility is estimated inappropriately highly (Piray and Daw, 2021). This implies that anxious individuals are insensitive to changes in true volatility and have impaired learning in environments involving low volatility - indeed, both of these effects have empirical support (Piray et al., 2019, Browning et al., 2015).

These two streams of findings, specifically that anxiety is associated with negatively-biased expectations of potential outcomes, and with impairments in adapting to environmental volatility, have begun to identify the computational mechanisms that might underlie pathologically anxious individuals’ altered reactivity to uncertain outcomes. The causal role of the anxious state (i.e. the anticipation for distal and uncertain threat) in the misestimation of uncertainty is unclear, but speculatively, these ‘impairments’ might have some adaptive value if an agent believes there may be incoming threat. Firstly, overestimating threat likelihood is adaptive as it is safer to overestimate than underestimate threat. Secondly, a bias to attribute environmental uncertainty to volatility rather than stochasticity allows an agent to respond more readily to changes in the environment – for example, an action which successfully avoided threat in the past may not be so effective in the present, which would be important to adjust for. Future work using induced- fear/anxiety designs will be important to understand if individual differences in threat estimation and dealing with uncertainty drive anxious states, or if indeed they are goal-directed changes in perception in line with threat avoidance.

## 5 Summary

We have presented an overview of the key themes of the computational literature on human fear and anxiety. Specifically, we discussed multiple computational processes involved in learning to predict and avoid threat, namely error-driven learning, the interactions of Pavlovian and instrumental learning and model-based planning. Given the diversity and complexity of these processes, the evidence suggests that there are multiple ways that learning can go awry with respect to pathological fear and anxiety, and different symptoms are associated with divergent computational mechanisms. We also introduced the few studies to date to study approach-avoidance conflict behaviour from a computational perspective, which have begun to suggest roles of decision uncertainty and asymmetries in learning about rewards and punishment, but there is not yet a clear computational account of approach-avoidance conflict. We also argued that uncertainty is a key motif implicated in anxiety, where converging evidence from different paradigms suggest that anxious individuals overestimate the likelihood of disadvantageous outcomes and struggle to learn in volatile environments. However, there are a number of challenges and opportunities for the future. Whilst computational approaches have been useful in deepening our understanding of the specific neurocognitive processes underlying defensive behaviour and how disruptions in these processes can lead to psychopathology, little progress has been made in informing treatment. Looking forward, computational approaches could be extended to better understand basic mechanisms and treatments for fear-and anxiety-related disorders. Further, better cross-species paradigms of defensive behaviour, especially those amenable to computational analysis (Redish, 2022), will be important in integrating findings across the human and animal literature and potentially spurring the development of psychiatric interventions (Pike et al., 2021) (Box 2).

Box 2 **Challenges and opportunities.**

**The factors driving pessimism under uncertainty.** There are multiple potential reasons why anxious individuals might be pessimistic during decision-making under uncertainty, or more specifically, accumulate negative evidence from the environment. This effect may be driven by individual differences in the representations of outcomes, for example by overweighting negative outcomes over positive ones (i.e. loss aversion) especially in fearful/anxious states, although this does not appear to fully explain the data (Charpentier et al., 2017). Alternatively, pessimism may be learned from one’s environment if negative outcomes historically occurred more frequently than positive outcomes. This leads to the question of whether pessimism can be unlearned, for example by exposure to an environment in which positive outcomes occur more frequently. Signal detection theory and Bayes’ theorem have been proposed as suitable frameworks to address these questions (Locke and Robinson, 2021, Huys et al., 2021).

**Translational approaches.** Some of the studies reviewed above have implemented translational behavioural paradigms to bridge the gap between the animal and human literature (Smith et al., 2021, Aylward et al., 2020). Computational approaches may be especially well-suited for translational studies of behaviour (Redish, 2022). The computations necessary to solve certain tasks (e.g. to avoid threat via reinforcement learning), even if these tasks are outwardly very different, may serve as a better criterion for translational validity as opposed to other criteria such as face or predictive validity, when the aim is to better understand the cognitive and neurobiological mechanisms underlying behaviour. This approach will be especially important for approach-avoidance conflict tasks, which are some of the most commonly employed rodent anxiety models (Campos et al., 2013). Future work should develop fear/anxiety tasks that are explicitly designed to engage similar computational processes in both animals and humans, which have been referred to as ‘common currency’ tasks (Pike et al., 2021). These tasks can also act as preclinical tests that will help to spur drug discovery for fear/anxiety disorders, which is especially important given that psychiatric drug development has slowed over the last decade (Hyman, 2012, Kesselheim et al., 2015).

**Computational mechanisms of anxiolytic/anxiogenic interventions.** Computational approaches are also well-suited for elucidating the cognitive and physiological mechanisms of anxiolytic and anxiogenic interventions. For example, traditional analyses based on summary-statistics of behaviour may demonstrate that drug X increased approach responses in an approach-avoidance conflict task, but this effect could be driven by changes in reward sensitivity or punishment sensitivity (or indeed both). Understanding pharmacological and psychological interventions at the level of cognitive/computational mechanisms will help to make treatments more targeted and therefore effective. A large-sample meta-analysis comparing pathologically anxious and depressed individuals to controls has already identified one potential computational target for therapy in punishment learning rates, where the learning behaviour of the symptomatic group was characterised by faster learning from punishment compared to the controls, but not sensitivity to punishment (Pike and Robinson, 2022). This suggests that treatment approaches in these patients could specifically target how they learn and adapt to negative events in the world, for example through cognitive-behavioural therapy (CBT) and this may improve treatment efficacy. Such approaches will be an important avenue for future research to potentially drive innovation in psychiatric treatment.

## Funding

This research was funded by a PhD studentship to YY from the 10.13039/100004440Wellcome Trust (222268/Z/20/Z) and a Senior Non-Clinical Fellowship to OJR from the Medical Research Council (MR/R020817/1). For the purpose of Open Access, the authors have applied a CC BY public copyright license to any Author Accepted Manuscript version arising from this submission.

## Footnotes

1 Although the term, emotional conflict, has been previously used to describe the interference effect of emotionally salient stimuli on cognitive processing (e.g. Bush et al., 2000; Etkins et al., 2006), the use of the term here reflects the degree to which individuals may be conflicted in approaching reward and avoiding affective (i.e. emotional) punishment. We use the exact name of the model parameter from the original study by Smith and colleagues.
